# A study of the factors influencing HIV-preventive intentions among “hookup” application users

**DOI:** 10.3389/fpsyg.2022.1048226

**Published:** 2023-01-04

**Authors:** Mengyu Li, Ning Li

**Affiliations:** ^1^College of Humanities and Development Studies, China Agricultural University, Beijing, China; ^2^Faculty of Modern Languages and Communication, Universiti Putra Malaysia, Serdang, Selangor, Malaysia; ^3^School of Journalism and Communication, Zhengzhou University, Zhengzhou, China; ^4^Department of Media and Communication, Kangwon National University, Chuncheon-si, South Korea

**Keywords:** social app, health belief model, planned behavior theory, hooking up, mindfulness, SEM model, HIV prevention

## Abstract

“Hooking up” refers to the act of experiencing sexual intimacy with strangers without committing to a romantic relationship. Social media provide more convenient conditions for hooking up; however, it also poses a greater risk for HIV infection. Therefore, it is necessary to study the factors influencing the HIV-preventive intentions of those who engage in online dating to devise effective strategies for preventing the spread of HIV. This study consisted of a questionnaire that was distributed to 520 users of the Hello Group application. The survey results revealed that structural equation modeling is a useful framework for understanding the risk of HIV transmission in casual hookup encounters. In addition, combining the health belief model with the theory of planned behavior can provide recommendations for enhancing HIV-preventive intentions among users of dating applications. The results showed that mindfulness, the Chinese cultural context, perceived benefits, and self-efficacy were the main predictors of users' HIV-preventive intentions when using online dating applications. Among the perceived HIV risks, only perceived barriers had a negative effect on users' HIV-preventive intentions. In addition, attitude, subjective norms, and behavior control served as mediating variables between independent variables and HIV-preventive intentions; however, the mediating effect of attitude on perceived benefits and intentions was not significant. According to our study, some users misunderstand the risks and make incorrect assessments of the cultural risks of hooking up. Therefore, it is necessary to pay attention to the research on the psychological tendencies of users and risk intervention when studying the concept of hooking up.

## Introduction

With the advent of the Internet and the growing popularity of smartphones, public acceptance of online dating has increased. Moreover, the way people express themselves emotionally and interact in close relationships is also evolving as a result of technological advancements. Currently, online dating applications (hereafter “apps”) are becoming popular mobile platforms in China, and Chinese people are engaging in “hookups” as a part of their social lives (Chan, 2020). Dating apps are becoming increasingly popular for people to meet each other for dating, sex, relationships, and more. There are now a variety of hookup apps, as they have become a common and routine form of social media. Using these apps allows people to create intimacy in surprising and complex ways (Petrychyn et al., 2020). Digital devices indeed enable novel methods for sex, intimacy, and sexual community, and they possess unique qualities and limitations (Race, 2015).

As the new generation of young people increasingly engages in digital dating, the penetration rate of online dating apps will continue to increase. However, many problems are emerging. One prominent problem is that dating apps have contributed to the spread of HIV. According to a United Nations study, the increasing rate at which young people are using mobile dating apps is a key factor in the recent resurgence of HIV among young people in Asia. The National Health and Family Health Commission of China recently released new data on HIV transmission. As of October 2021, the total reported HIV incidence was 5,357, and the number of AIDS-related deaths was 1,849 in China (National Health Commission of the People's Republic of China, 2020). At the end of 2020, 1.053 million people were living with HIV/AIDS nationwide, and 131,000 new cases were reported in 2020. In the past decade, the number of new cases reported nationwide each year has increased year over year; however, the growth rate declined in 2020 due to the impact of COVID-19. The number of patients currently living with HIV or AIDS has exceeded 1 million, and there are approximately 100,000 new cases every year (Chinese Center for Disease Control and Prevention, 2021).

In China, HIV is mainly transmitted sexually, with 95% of cases resulting from sexual transmission. The majority of transmission occurs between heterosexual couples, and the infection rate among men is higher, which is related to the fact that men more often have multiple partners, engage in online dating, and are more sexually active. Regarding the age of those infected, China's HIV incidence has displayed a trend of “rising at both ends.” In 2020, the majority of HIV patients over 60 were men. There were a total of 23,976 men over 60 with HIV, accounting for about 18% of the total number of cases.

Moreover, among all cases, heterosexual transmission accounted for 93.8%, and homosexual transmission accounted for 4.7%. There were about 3,000 young students aged 15–24 years with HIV, accounting for 22.3% of all HIV cases among young people. Moreover, 81.7% of these transmissions were from homosexual sexual activity, while 16.9% resulted from heterosexual contact.

“Hooking up” refers to the act of randomly selecting a sexual partner using some sort of social networking app on the Internet, and it involves going on a date to engage in sexual behavior and obtain sexual satisfaction. Research showed that the intimate publicity available to female users of hookup apps is broader than that afforded by in-app interactions; there is an entire network of intimacy, sociality, and publicity that has been found around hookup apps (Petrychyn et al., 2020). According to survey data, the incidence of traditional sexual behavior among college students in China is 15.1%. In the same population, the incidence of engaging in sexual behavior *via* online dating is 11%. Because college students seek to satisfy their curiosity and sexual needs, they are more likely to engage in unsafe sex because of their pursuit of novelty, adventure, excitement, and complex sensory experiences (Ren et al., 2022). The secrecy and anonymity of online social networks gradually weaken college students' moral restraint and self-control, which promotes hookups *via* the Internet and in turn increases the risk of HIV transmission. College students have a weak sense of self-protection and lack awareness of sexual safety. Although online dating applications may satisfy users' desires for love and sex, the associated risks are often overlooked (Solis and Wong, 2019). Penhollow et al. (2007) asserted that users' involvement in casual, high-risk sexual encounters facilitated by dating apps exposes college students to sexually transmitted infections and unintended pregnancy. The changing social contexts of HIV are reflected in the evolving phenomenon of the increase in “men who have sex with men” (MSM) in the Philippines who seek out partners online *via* social networking apps (Hollingshead et al., 2020). The administrators of several dating apps have realized the risk of HIV transmission. For example, Blued carried out mobile HIV testing, publicity campaigns, consultation sessions, data-driven studies, and antidiscrimination advocacy; it has also explored a new model of “Internet + HIV prevention and control.” Giles (2021) reported that location-based mobile dating applications are often a rich source of personal information readily accessible to strangers online. In many cases, this information now includes users' HIV status and the date of their most recent sexual health test.

As dating apps continue to evolve and acquire new characteristics, the number of users is also growing. Moreover, people are increasingly using dating apps to meet each other in real life, leading to an increased risk of HIV transmission. According to our survey, the number of “stranger” social app users in China is still rising. Dating apps have become a prominent and contentious topic in discussions over intimacy among the wider public and in academia (Wu and Trottier, 2022). Chan (2020) conducted a detailed empirical study of the multiple uses of Momo, a popular Chinese dating app. It is difficult to discern whether Momo is an alternative to the new liberalization of intimate relations in China. Therefore, studying the impact of arranging real-life meet-ups *via* dating apps is necessary. Based on existing knowledge in the field, this study attempted to compile in-depth analyses of the relationship between hookup culture and HIV-preventive intentions through dating apps. Dating apps mediate users' dating and sexual practices, as well as their attitudes toward HIV.

After considering the number of users of each online dating app, as well as users' daily activities and their age distribution, we decided to recruit users of the social app Hello Group to participate in a questionnaire with the goal of better understanding users' cognition and influence on online social behavior. Hello Group is an open, video-based smartphone application that was launched in August 2011 and is based on the location of the user. Hello Group dominates the social field of strangers in terms of total users and monthly active users; it has a wide range of users, from teenagers to people in their 70s and 80s. In terms of total monthly use time, Hello Group is a leader. However, Hello Group's penetration rate remains relatively low, indicating that this app still has a significant amount of work to do to attract users.

The rise in dating apps has led to an increase in online dating, which in turn has exacerbated the spread of HIV and other sexually transmitted diseases (Yu, 2021). This has undoubtedly raised the need for the developers of such dating apps to establish norms and remind risks regarding the usage of these apps. In a sense, hooking up in China is not based on a fixed system, which is only maintained by a short sense of freshness and excitement. Simultaneously, due to a lack of effective means of auditing strangers' dating apps, it is hard for users to get the risk reminder on platforms, which increases the risk of the transmission of HIV and other sexually transmitted diseases.

This study combined the health belief model (HBM) with the theory of planned behavior (TPB), which is the successor to the theory of rational action (TRA), to describe the users of online dating applications in China through structural equation modeling (SEM). It also analyzed the relationship between users' mindfulness, perceived benefits, perceived barriers, self-efficacy, and HIV prevention intentions; it also considered whether attitudes, subjective norms, and behavior control play a mediating role between independent and dependent variables. This study might serve as an important reference for subsequent research and prove crucial to preventing the spread of HIV. Through a case study of the popular Chinese online dating app Hello Group, this study examined an understudied aspect of online dating apps: (1) the types of sexual activities that these apps tend to encourage, facilitate, and mediate; (2) the hookup culture that has emerged on social media websites and applications as well as the perceptions surrounding HIV risks.

The literature review section of this paper summarizes existing research on hookup culture and HIV, the HBM and TPB, both within China and abroad, and related research on mindfulness. The third section introduces the research methods used in this study. The fourth section uses SPSS and SEM to analyze the statistical data from the questionnaire before analyzing the influencing factors on HIV-preventive intentions among online dating app users. The final section of the paper is the conclusion, which discusses this study's results, contributions, and limitations; it also discusses possibilities for future research.

## Literature review

Research on hookup culture in Western countries began relatively early, and previous studies examined the relationship between hookup culture, health, and romance, as well as the associated risks. They have connected with dating apps to undertake research. Paul et al. (2000) defined “hooking up” as a form of sexual contact that may or may not include sexual intercourse. Additionally, hooking up implies a single event between two strangers or people who have met briefly. Their study used various social and psychological predictors to explain the differences between different hookup experiences among college students. Fielder et al. (2013) believe that “hookup behavior” refers to sexual interactions between partners who are not in a relationship and do not anticipate mutual commitment. Their study focuses on whether the hookup culture will replace normal romantic behaviors. Through the lens of social theory, Anders et al. (2020) observed that hookup behavior can bring people enjoyment, sexual fulfillment, status, achievement, and the potential to develop a relationship; however, it also brings corresponding costs, such as regret, ambiguity, increased sexual risk, and loss of respect. Garcia et al. (2012) studied various factors affecting hookup behavior, and after reviewing previous studies related to hooking up, they found that hookup behavior is deeply rooted in popular culture.

Moreover, Kalish and Kimmel (2011) believe that hookup culture is strongly related to gender and emphasize the positive impact of hookup culture on young women's social interactions in the United States. Montes et al. (2017) studied the positive correlation between the attitudes of relatives, friends, and participants toward hookup behavior. Scholars exploring digital intimate publics tend to consider social media platforms separately from dating and hookup apps, implying that there is a distance between social and sexual communication practices (Byron et al., 2021). Participants' attitudes toward hookup behavior were positively correlated with their social motivation to hook up. This increase in social motivation is positively correlated with hooking up with multiple partners and its negative consequences.

Media and communication researchers studied dating apps across different cultural contexts to better understand the dynamic relationship between dating apps and social processes. Liu (2016) considers how sexualities can constitute a useful lens for understanding social media in order to clarify the complex interconnections between the political, cultural, economic, and “private” realms of sexual experience. Moreover, Liu T. et al. (2022) investigated how rural migrant workers in China use digital dating services and select daters, revealing that, when dating online, there are many obstacles to achieving sexual and romantic satisfaction. In addition, Albury et al. (2020) identify a significant category of supportive discussions of safer app use within social news and lifestyle reporting and they also reveal app users' safety strategies, and their experiences of pleasure and playfulness. Furthermore, Conner (2019) demonstrated how gay men's portrayals of themselves on dating apps perpetuate biases based on body type, age, race, and the HIV stigma.

Similarly, Winter et al. (2020) proposed that it is important to understand the psychosocial variables related to sexual behavior, especially among members of high-risk groups, such as those who engage in hookups; they also found that HIV screening, which is a behavior related to sexual health, is influenced by the body image of those who have sex with others through apps. However, Hollingshead et al. (2020) found that the participants in their study viewed the expanding epidemic and apps as intimately linked and regarded apps as “risky spaces” for “risky behavior.” Additionally, Lauckner et al. (2019) claimed that dating apps can be detrimental to people because of their potential to create negative and traumatic experiences.

This study was based on the HBM and the TPB. Therefore, this study also surveyed the relevant literature. The HBM and the TPB are two widely used theories in the field of health psychology. Because both theories are based on expected value theory and because the concepts intersect with each other, they form a complementary relationship. Therefore, many studies that seek to interpret and predict health-related behaviors have comprehensively applied both theories to improve their accuracy of interpretation. Nothling and Kagee (2013) attempted to determine whether the main components of the health belief model—perceived susceptibility, perceived severity, perceived benefits, and perceived barriers—can predict the acceptance of routine HIV counseling and testing and whether action cues can predict the adoption of routine HIV counseling and testing. Smith et al. (2012) studied the empirical verification of the behavioral health model for HIV risk. Fan et al. (2018) found that behavioral intervention can change health beliefs and that such intervention may make people more willing to accept AIDS testing. Buldeo and Gilbert (2015) explored the HBM and the willingness of 1st-year students at the University of South Africa to voluntarily seek consultation and testing for AIDS. The investigation concluded that knowledge about HIV and AIDS and voluntary counseling and testing are crucial to HIV management and prevention. It also found that college students' self-efficacy and action tips may have a positive effect on AIDS prevention. Huang and Pan (2013) studied the predictors of female sex workers' willingness to prevent HIV exposure in southwest China. They assessed their understanding of AIDS, its psychosocial impacts, demographic data, and their willingness to be tested for AIDS.

The HBM refers to the behaviors and belief prevention adopted by individuals, including the knowledge of the disease and health knowledge, to maintain and promote health and achieve self-satisfaction and self-realization. The HBM is based on the needs and motivation, cognitive, and value expectation theories. It is concerned with people's attitudes and beliefs toward health and accords importance to the internal and external factors that affect those beliefs. The HBM was the first theory to explain and predict health behaviors. It was proposed in 1952 by three social psychologists, Hochbaum, Rosenstock, and Kegels. The HBM asserts that individual perception, positive action, and the belief that one can take recommended actions are important factors for behavioral change. The model is used to explore various long- and short-term health behavioral problems, including sexual risk behaviors and the spread of HIV and AIDS. The HBM consists of three parts: individuals' health beliefs, the clues or intentions of behavior, and behavior constraints. Individuals' health beliefs refer to how people view health and diseases, how they understand the severity and susceptibility of disease, and how they understand the effect of preventive measures and the obstacles encountered when taking such measures. According to the HBM, if people want to accept the advice of medical staff and adopt healthy behaviors or give up harmful behaviors, they need to meet several conditions:

1) Individuals must be able to perceive the threat of a disease or risk factor and subsequently recognize the seriousness of the problem, including disease susceptibility (perceived susceptibility) and the perception of disease severity (perceived seriousness). They must be able to estimate the consequences of adopting or abandoning a behavior, which includes understanding the benefits of the behavior (perceived benefits) and the barriers to implementing or abandoning such behaviors (perceived barriers). They must also have efficacy expectations, which refer to one's ability to implement or abandon a behavior, also known as self-efficacy. Furthermore, Chen et al. (2020) claimed that social support, self-efficacy, and apps are employed in a variety of situations. An app that takes the promotion of social support and self-efficacy as its core and transforms it into a game-based interaction method to achieve the goal of sustainability is more valuable (Chiou et al., 2021).
2) They must possess clues or action intentions, which refer to the factors determining whether people will take preventive measures (Chiou et al., 2022) based on media reports, reminders from medical staff, advice from experts, and relatives' and friends' experiences with the disease.
3) They must be able to constrain their behavior based on demographic characteristics (age, gender, race, place of origin, and so on), social psychological factors (personality, social class, the influence of peers and others, and more), and knowledge structure factors (knowledge about the disease, previous experience with the disease, and so on.).

**Figure 1:**
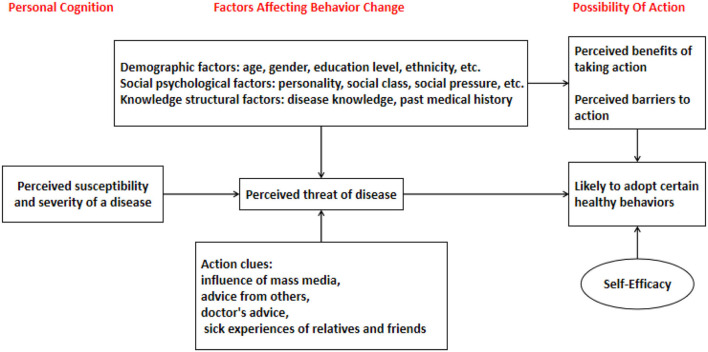
Health belief model framework.

The framework of the HBM is shown in Figure 1.

The TPB was proposed by Ajzen (1988, 1991). This theory is the successor of the TRA, which was jointly proposed by Ajzen and Fishbein (1975, 1980). Because Ajzen's study found that human behavior is not completely voluntary but rather controlled, he expanded on TRA, adding the new concept of “self-behavior control cognition.” Subsequently, TRA developed into a new behavioral theory known as the TPB. The TPB has five elements: attitude, subjective norms, perceived behavioral control, behavior intention, and behavior. Among them, *attitude* refers to an individual's positive or negative feelings about a behavior. In other words, an attitude is formed as a result of the conceptualization of an individual's evaluation of a specific behavior.

**Figure 2:**
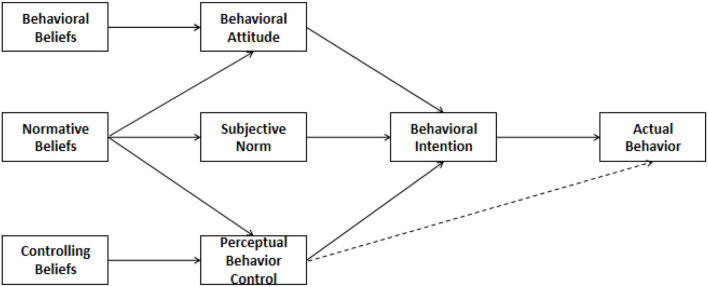
The theoretical framework of planned behavior.

Therefore, the components of attitude are often regarded as a function of an individual's significant belief in the result of a behavior. Subjective norms refer to the social pressures that individuals feel when deciding whether to resort to a certain behavior. In other words, it refers to the influence of an individual or group on an individual's decision regarding whether to engage in a certain behavior when predicting others' behavior. Perceptual behavior control refers to the obstacles that arise from individuals' past experiences and expectations. Individuals believe that the more resources and opportunities they have and the fewer obstacles they meet, the stronger their perceptual behavior control will be. It can influence behavior in two ways: First, it has motivational implications for behavioral intention. Second, it can directly predict behavior. Behavioral intention refers to an individual's judgment of the subjective probability of taking a specific action, which reflects an individual's willingness to take that action. Behavior refers to the behaviors in which an individual engages. Ajzen (1991) believes that all factors that can affect behavior also indirectly affect behavior through behavioral intention. According to the TPB, behavioral intention is affected by three related factors. The first factor is the “attitude” of the individual toward adopting a specific behavior. The second is the external “subjective norm” that will drive an individual to take a specific action. The third is “perceived behavior control.” The theoretical framework of planned behavior is shown in Figure 2.

Only a few studies combined the TPB and the HBM to investigate health and health-related behaviors. McClenahan et al. (2007) tested the utility and efficiency of the TPB and the HBM in predicting testicular self-examination behavior, and they found that self-efficacy was the most important predictor of testicular self-examination behavioral intention in both models. The TPB and the HBM have the potential to both improve our understanding of adherence behaviors and contribute to the design of more effective interventions to promote adherence to TB and HIV/AIDS medications (Munro et al., 2007). Reid and Aiken (2011) combined key concepts from five health behavior models, including the TPB and the HBM, to predict condom use intention. Seong and Bae (2022) investigated adults' health behaviors in relation to pandemic prevention based on the HBM and the TPB. Bish et al. (2000) used two social cognition models, the HBM (Maiman and Becker, 1974) and the TPB (Ajzen, 1991), to report on a study that identified predictors for 142 women receiving cervical screenings in central London. However, most studies linking the two models have focused on comparing the utility of the two models in predicting behavioral intentions, and they have found that the TPB tends to be superior (Bish et al., 2000; McClenahan et al., 2007).

Although the TPB and the HBM emphasize different aspects of behavior formation, they have some natural linkages. Lawes-Wickwar et al. (2021) investigated the effect of perceived disability on pandemic disease prevention behavior, attitudes toward pandemic prevention behavior, subjective norms, and perceived behavioral control. Yang (2015) compared the predictive power of the TPB, the HBM, and an integrated model and found that several of the HBM variables influenced behavioral intention through the TPB variables. In addition, possible mediating and moderating effects among variables were examined to simplify the direct and indirect effects of key variables on behavioral intentions (Yang, 2015). Findings from a study by Seong and Bae (2022) suggest that the TPB and the HBM are pursuing essentially the same fundamental constructs that account for behavioral formation. These findings are expected, given that both models are based on expectancy-value theory (Bish et al., 2000). Both models recognize the influence of self-efficacy on individuals' decisions to adopt healthy behaviors. In other words, behavior control is an important factor that leads to the initiation of behavior. Moreover, the subjective norms of the TPB may impact the subjective norms of the HBM's cues to action that trigger healthy behavioral changes (Yang, 2015). Particularly, even when exposed to external stimuli in one's social environment, people who are prompted to take precautions are more likely to conform to social norms. Garcia and Mann (2003) explained 19% of breast self-examination behavioral intention using the HBM. Self-efficacy and perceived control over behavior are frequently the most important predictors of health behavior intentions and behavior (Manstead and Van Eekelen, 1998; Armitage and Conner, 1999). The classic mediation effect in Yang's study explains why the interpersonal discussion was no longer significantly related to behavioral intention after subjective norms were put into the model (Yang, 2015). Thus, subjective norms are expected to mediate the relationship between action cues and behavioral intentions.

Recent theoretical developments have also demonstrated a connection between the TPB and the HBM. In particular, communication scholars emphasize that cost-benefit analysis should be an integral part of the conceptualization and evaluation of attitudes within the TPB (Fishbein and Yzer, 2003). Ajzen (1988) suggested that attitude, which is an extension of behavioral beliefs, should contain both instrumental and experiential attitudes toward a behavior. Similarly, the findings of some scholars provide more evidence that supports claims for more studies on response costs, which include concerns about the possible negative consequences of implementing health behaviors (Champion and Skinner, 2002; Cameron et al., 2009). Bish et al. (2000) suggested that the poor prediction of intention provided by the HBM was due to a lack of correspondence between measures of the HBM variables and measures of intention. This was followed by a cost-benefit analysis, and the potential outcome is that researchers can identify the behavioral beliefs underlying a person's decision to adopt recommended behaviors (Yang, 2015). Therefore, attitude can influence the relationship between perceived benefits and dependent variables. Fishbein (2007) argued that many behavioral determinants, such as perceived threats and benefits, are reflected in behavioral, normative, and control beliefs. They serve as antecedents to attitude, subjective norms, and perceived behavioral control. The perceived ability to control one's behavior was found to have both direct and indirect effects (Seong and Bae, 2022). Gi theory development, the indirect effects identified through path analysis and additional mediation and moderation tests suggest that most of the HBM variables might be indirectly related to behavior because their relationship with behavioral intentions was either mediated or moderated by the TPB variables (Seong and Bae, 2022). Yang (2015) explored the links between the HBM and the TPB, with a particular focus on how the HBM variables can be used as antecedents for the existing TPB variables to enhance the overall predictive power of the model.

With the rising popularity of the Internet and the emergence of dating apps, there has been an increase in research on dating behavior on dating apps across the globe. Popular media outlets have described intimate relationships among contemporary college students as dominated by a pervasive sexual “hookup culture,” suggesting that students are involved in frequent sexual encounters without expecting a continuing relationship. This hookup culture has been described as “a nationwide phenomenon that has largely replaced traditional dating on college campuses” in the USA (Bogle, 2008; p. 5). Dai (2021) investigated dating apps such as Tinder to study the relationship between smartphones and college students' sexual health and relationship experiences, including sexual attitudes, changes in relationships, and dangerous sexual behaviors. The growing use of dating apps will influence people to be more accepting of sexual indulgence, resulting in more risky sexual behaviors and higher relationship turbulence. Chan (2018) believes that the popularity of mobile dating apps has changed how gay individuals communicate, and he links this online intimacy with online individualism and neoliberalism. Pan and Huang (2012) randomly sampled.

Chinese people aged 14 to 61 years. Their study constituted the first use of data to demonstrate the incidence and linear regression relationship between various situations of online sex among different social classes, thus proving that online sex is a new, mainstream form of culture. Qiu and Huang (2020) believes that the widespread popularity of mobile dating has greatly liberated male sexuality from the orthodox concept of heterosexuality; however, the increase in dating opportunities will also expose this group to a higher risk of contracting AIDS. Some relevant risk-coping strategies include actively choosing sexual partners, building trust, enhancing one's perception of health risks, and adopting safe sexual habits. Li and Wu (2018) studied the engagement behaviors of college students from the perspective of self-rationalization through interviews with 18 college students. They analyzed the relevant factors for the rationalization of the occurrence and continuation of the engagement as well as the resolution of the engagement, such as physiological and emotional needs, conformity psychology and peer pressure, primary family and major events in childhood, failure to establish romantic relationships, psychological contradictions, and moral confusion of the parties. Tang and Dong (2017) believe that meeting strangers through social media is a strong temptation facing youth; they also assert that the related risks, such as being cheated on and engaging in one-night stands, are substantial. There is an urgent need to guide, educate, control, manage, and standardize the use of social media for meeting strangers. Xu and Wu (2019) discussed a phenomenon that has become popular in mainland China in recent years: “stranger communication.” They paid special attention to an app called Momo, a social discovery and dating platform widely used in China.

In addition, this study explored the impact of mindfulness and traditional Chinese culture on hookup behavior. Mindfulness is defined as bringing one's attention to the experience of the present moment with an attitude of acceptance, and it is associated with engagement in various health behaviors. The term “mindfulness” describes a state of awareness in which one is fully present in the moment without analyzing, judging, or reacting to anything. In other words, mindfulness is the act of simply perceiving things and paying attention to them. Kabat-zin defined mindfulness as a purposeful and conscious focus on the present without judging the present (Kabat-Zinn, 2003). Mindfulness-based stress reduction aims to help people eliminate negativity bias by amplifying intention and minimizing judgment (Liu et al., 2022a). Mindfulness training teaches one not to treat life stress as a difficulty or disaster but rather as an adjustable method for relieving emotional stress (Fernandes et al., 2021). Previous research showed that mindfulness can have a positive impact on different populations. For example, increasing mindfulness among clinicians can improve the safety competence of medical staff (Braun et al., 2019), patients (Liu et al., 2022b), employees (Liu et al., 2022c), and flight attendants (Liu et al., 2022d,e). It has also been demonstrated that mindfulness is an effective method for coping with COVID-19-related stress (Weis et al., 2021). According to Liu et al. (2022a) and Chen et al. (2022c), mobile health overcomes many obstacles associated with traditional mindfulness meditation training. Some studies discussed users' perceptions of HIV prevention using the HBM (Liu H. et al., 2022) or health behavior changes based on the intervention of mindfulness (Asfar et al., 2022). However, few studies explored the effects of dating apps from a perspective that combines mindfulness with the HBM or the TPB. This study attempted to fill this literature gap by employing systems thinking.

Although studies indicate that individual differences in mindfulness do not reliably translate into a pattern of healthy behaviors, mindfulness shows a stronger association with healthy behaviors under certain conditions (Sala et al., 2020). Mindfulness-based stress reduction leads to increased problem-solving styles, life satisfaction, and increased regulation of negative emotional stimuli while also reducing aggression among HIV-positive young people (Webb et al., 2018). Sala et al. (2020) clarified that the importance and utility of mindfulness in improving health behaviors might be limited in the general population. However, mindfulness had relatively stronger associations with health behaviors in some populations, suggesting that there are important limitations to the associations between mindfulness and health behaviors. Wedell et al. (2022) studied the buffering role of mindfulness in the relationship between sexual orientation, affective lability, and suicidal ideation.

Additionally, they found that several mindfulness facets significantly buffered the indirect relationship between sexual minority identity and suicidal ideation *via* affect liability. Chen et al. (2021) explored and examined the effects of loving-kindness meditation on doctors' mindfulness, empathy, and communication skills. They suggested that the mechanisms that underlie the effects of loving-kindness meditation on mindfulness, empathy, communication skills, and other psychological constructs need further elucidation. Moreover, Chen et al. (2022a,b) reported that focused-attention meditation could significantly improve surgeons' focus, communication skills, and safety attitudes, potentially helping to reduce the frequency of adverse clinical events. In addition, as a simple and effective intervention technique, mindfulness meditation improves patient safety and has a certain promotional value (Liu et al., 2019).

Online dating app users exist “in-between” traditional Chinese culture and new values. They are contributing to the formation of a new form of Chinese cosmopolitanism by cultivating insensitivity toward strangers. Moreover, their participation in unfamiliar modes of communication means that those who engage in online dating are more open to others. Xu and Wu (2019) adopted cultural discourse analysis to analyze the results of online and offline interviews conducted in Beijing and Shanghai. They investigated how Momo users in urban metropolises use the application and analyzed cultural radiation in their communication practices. Online dating apps have impacted contemporary intimacy. One popular app, Bumble, claims to be “shifting old-fashioned power dynamics” by requiring women to “go first” in conversations with “matched” men (Young and Roberts, 2021).

Additionally, Chen et al. (2021) examined the digital dating experiences of members of the Chinese diaspora in Australia who use Chinese- and English-language online dating apps and hookup services. They found that online dating and its constitutive norms play a role in maintaining certain tropes of cultural representation regarding racial subjects and in securing cultural power within an overarching system of white racial entitlement. Hookup culture is consistent with larger cultural shifts in the “scripts” and terminology surrounding sexuality (Monto and Carey, 2014). Moreover, Liu T. et al. (2022) demonstrated that, while online dating increases a sense of possibility and desire in China, particularly among individuals of lower socioeconomic status, it fails to support these users in tackling the structural inequalities that obstruct the realization of their desires. If culture is a “toolkit” offering culturally competent actors a set of ideas and practices with which to explain their choices, for instance, to use Ann Swider's metaphor from her article “Culture in Action,” then the hookup culture offers students many tools for embracing casual sex but few for articulating why they may prefer other kinds of sexual engagement, or none at all (Wade, 2017). In addition, Xiong and Liu (2022) believe that the ways in which the “super-sticky” “all-in-one platform” WeChat acts as the coordinator of a polymedia environment - and not just part of the polymedia environment - in mediating intercultural romantic relationships in the Greater Bay Area of China. However, another study studied online dating platforms as sites. These researchers focused on examining people's quotidian and habitual engagements with online dating platforms, and they perceived them as significant areas for the exercise and negotiation of “new” rules for intimacy (Liu, 2019). However, this study not only examines online dating platforms as hookup group active communities but also focuses on what factors influence hookup app users' perceptions of HIV and whether the establishment of intimate relationships is affected by mindfulness, the Chinese cultural context, and other factors.

Even in the modern era, most young people still fall in love gradually, with many platonic friendships and school relationships progressing to romantic ones. Many people are still hesitant about hooking up and avoid talking about it. In addition, HIV is regarded as a disgrace because it is related to immoral behaviors, such as drug use or sexual promiscuity. However, with the influence of Western culture, young people's acceptance of hookup culture is constantly increasing. This increases the number of dating app users, leading to more hookup behavior. This study summarized the key risk and vulnerability factors for HIV infection and transmission associated with hookup culture through online dating apps and proposed directions for future research.

## Research method

**Figure 3:**
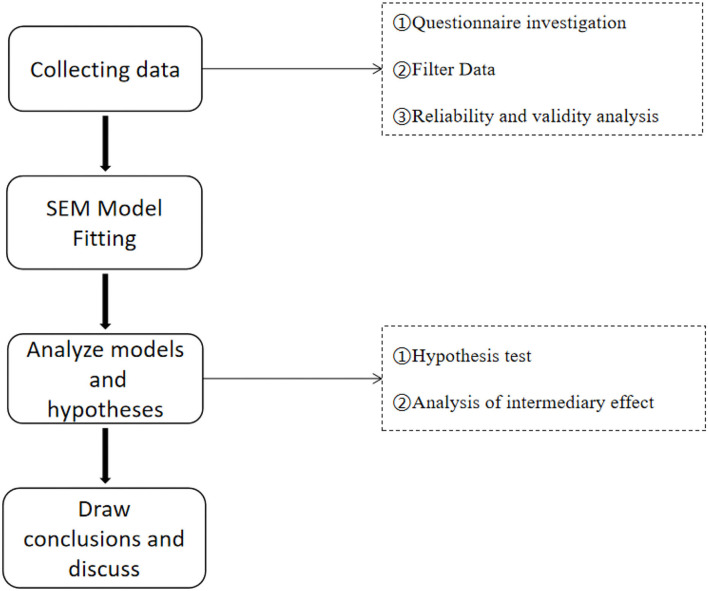
Research flow chart.

This study collected data on people who engage in hookup behavior using questionnaires. After screening the data, reliability and validity analysis methods were used to ensure the accuracy of the data. In addition, the SEM model was fitted through Amos and hypothesis testing, and intermediary effect analysis was conducted to verify the model's accuracy. Subsequently, we developed relevant hypotheses. The research flow chart is shown in Figure 3.

The questionnaire collected information on users' gender, age, location, sexual orientation, and current emotional status. It also collected information on whether a participant was a talk-to-strangers app user, whether they had used other dating apps, which dating apps they preferred, and their main reasons for using them, whether they believed dating through a dating app was reliable, whether they were the active or passive party when dating, whether they used their real information on the dating app and their perspective on online dating behavior through dating apps, whether they took safety measures and their perception of the consequences of online dating behavior, whether they thought dating apps contributed to the spread of AIDS, and what measures they though could be taken by dating apps to prevent the spread of AIDS. These questions were designed to obtain a comprehensive understanding of the users of dating apps, their characteristics, and hookup behaviors.

The questionnaire survey method offers high efficiency, objectivity, and universality. Because Chinese people tend not to reveal that they engage in hookups and because questionnaire surveys were universal, this study used a quantitative research method to study the relationship between users' mindfulness, culture, perceived benefits, perceived barriers, self-efficacy, and HIV prevention intentions. The online questionnaire survey was carried out using the online survey website. Questionnaire Star, and a 5-point Likert scale was used. Questionnaire participants had to answer several questions, and they were given the options of “totally agree,” “agree,” “doesn't matter” (uncertain), “disagree,” and “totally disagree.” The researchers joined the group chat of the Hello Group app and distributed links to users of the Hello Group app. The link to the questionnaire was valid for 2 months. In all, 600 questionnaires were distributed, 535 were collected, and 520 valid questionnaires were finally obtained after the screening was conducted. Subsequently, the reliability and validity of the data obtained from the survey were analyzed. This study did not collect any private user information in the data collection process.

**Figure 4:**
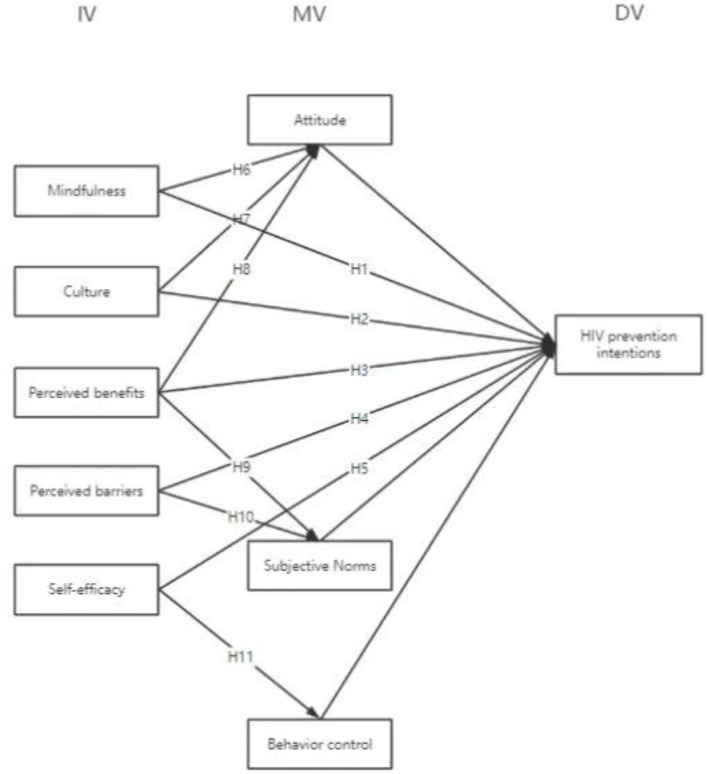
Structural equation modeling.

Our literature review demonstrated that most existing studies are case studies that employ qualitative methods. This study focuses on measuring data, establishing models, and testing hypotheses. Therefore, this study adopts a quantitative approach. Based on the HBM and the TPB, this study uses SEM to model and then does fit analysis. Based on the literature review and questionnaire methods, this study aimed to profile the users of hookup apps in China and influence their HIV risk perception and prevention intentions. The basic framework of the model used is shown in Figure 4.

This model positioned mindfulness, culture, perceived benefits, perceived barriers, and self-efficacy as the independent variables. The dependent variable was HIV-preventive intentions, and the control variables were demographic factors. Demographic factors such as gender and age might affect the final result, making it impossible to determine whether other independent variables influenced the final result. Therefore, demographic factors were considered control variables. In addition, users' attitudes, subjective norms, and behavior control could not be manipulated and controlled in advance. Moreover, because the aforementioned aspects were internal factors that could not be directly observed, they were considered mediating variables of the model.

**Figure 5:**
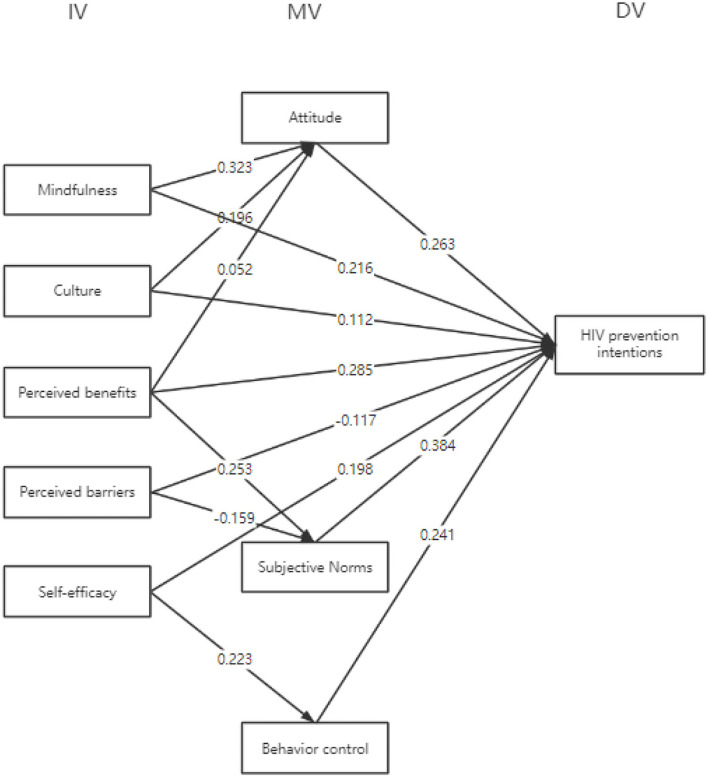
SEM model fitting for prediction of HIV prevention intention in users.

To classify the problems, we established mindfulness, culture, perceived benefits, perceived barriers, and self-efficacy as the five independent variables. Attitude, subjective norms, and behavior control were the mediating variables. Demographic factors served as the control variables, and HIV-preventive intentions were set as the dependent variable. The SPSS and Amos were used to obtain a fitting SEM model. The results are shown in Figure 5.

The fitting analysis of the SEM model was carried out. Table 1 illustrates the specific analysis results and parameters.

**Table 1:** SEM model fit analysis.

**Fit metrics**	**CMIN/ DF**	**RMSEA**	**TLI**	**NFI**	**IFI**	**CFI**
Value	2.285	0.059	0.953	0.914	0.952	0.954
Standard	< 3.0	< 0.1		>0.9	>0.9	>0.9

The above table indicates that the hypothetical model and data match well (CMIN/DF = 2.285, TLI = 0.953, NFI = 0.914, IFI = 0.952, CFI = 0.954, and RMSEA = 0.059).

This study considered the physiological and psychological needs, cognition, and value expectations of the participants in the network engagement. For this purpose, we presented the following hypotheses:

H_1_: Mindfulness has a positive and significant impact on users' HIV-preventive intentions.
H_2_: Culture has a positive and significant impact on users' HIV-preventive intentions.
H_3_: Perceived benefit has a positive and significant effect on users' HIV-preventive intentions.
H_4_: Perception barriers have a significant negative impact on users' HIV-preventive intentions.
H_5_: Self-efficacy has a positive and significant effect on users' HIV-preventive intentions.
H_6_: Attitude has a significant mediating effect on users' mindfulness and HIV-preventive intentions.
H_7_: Attitude has a significant mediating effect on culture and users' HIV-preventive intentions.
H_8_: Attitude has a significant mediating effect on users' perceived benefits and HIV-preventive intentions.
H_9_: Subjective norms significantly mediate users' perceived benefits and HIV-preventive intentions.
H_10_: Subjective norms have a significant mediating effect on users' perceived barriers and HIV-preventive intentions.
H_11_: Behavioral control has a significant mediating effect on users' self-efficacy and HIV-preventive intentions.

Although researchers carried out a variety of in-depth studies on the popularity of dating apps, the spread of online dating, and the associated risks of AIDS transmission, there remains a lack of research on the generation mechanisms and influencing factors of online dating in the specific cultural and social contexts of China. The hidden influencing factors, intermediary effects, and regulatory effects of online dating are unknown research topics that require further exploration. Only through sufficient investigation and research can scholars more clearly understand how users of dating apps recognize their attitudes toward online dating, the risks of online dating, and HIV-preventive intentions. This study will help us better understand the motivations behind choosing to engage in online dating and its physical and psychological impact. Therefore, the framework model comprehensively considered the influencing factors of HIV prevention intention among online dating app users.

## Results and discussion

### Direct effects

#### Demographics

First, an analysis was done of the demographic factors in the questionnaire, including age, gender, location, wealth index, education level, and occupation (see Table 2 and Figure 4 for the analysis results of the questionnaire).

**Table 2:** Questionnaire participants' gender and age statistics.

**Age range**	**Gender**		**Proportion**

	**Men**	**Women**	
< 18	13	9	4.23%
18–29	99	74	33.27%
30–45	79	86	31.73%
45–60	61	87	28.46%
>60	5	7	2.31%

Because this study primarily explored the impact of mindfulness, culture, perceived benefits, perceived barriers, and self-efficacy on HIV prevention intention, it is uncertain whether age and gender will affect the study results. Therefore, to control for the impact of uncertainty, demographic factors, such as gender and age, were analyzed as control variables.

Table 2 illustrates that young people aged 18–29 years accounted for 33.27% of the survey participants, and people aged 30–45 years accounted for 31.73% of the participants. This demonstrates that users of dating apps are young and middle-aged. Moreover, the number of participants over 45 years old was 28.46%, highlighting the need to pay attention to middle-aged and elderly users.

The results show that most of the participants who responded to the questionnaire were between 18 and 60 years old, and the proportion of men to women was relatively unequal. The statistical data exhibited normal distribution characteristics. In addition, the regional distribution of questionnaire participants was also considered. Except for Tibet, Xinjiang, and Taiwan Province, there were participants from all regions of China. Among them, Anhui, Jiangsu, Liaoning, and Fujian provinces had the largest number of participants, which indicates that the users participating in this survey were widely distributed and representative.

The pilot study questionnaire's reliability test can determine the sample data's consistency and reliability. The Cronbach's Alpha value of each variable and each dimension is greater than 0.800, and the Corrected Item-Total Correlation (CITC) value is greater than 0.5. Therefore, each variable in this study is greater than 0.5. The reliability coefficients of its measurement dimensions are all within a reasonable range, indicating that the questionnaire has high consistency and stability, indicating that the reliability results of the pilot study are ideal. Results of reliability test are indicated in Table 3a.

**Table 3a:** Reliability test results.

**Variable**	**CITC**	**Cronbach's alpha**
Mindfulness	0.736	0.933
	0.751	
	0.764	
	0.743	
	0.803	
	0.765	
	0.809	
	0.768	
Culture	0.736	0.909
	0.707	
	0.769	
	0.664	
	0.801	
	0.819	
Perceived benefits	0.602	0.879
	0.726	
	0.735	
	0.680	
	0.820	
Perceived barriers	0.592	0.827
	0.592	
	0.602	
	0.600	
	0.726	
Self-efficacy	0.787	0.927
	0.796	
	0.810	
	0.699	
	0.825	
	0.808	
Attitude	0.811	0.907
	0.762	
	0.678	
	0.813	
	0.766	
Subjective Norms	0.622	0.868
	0.586	
	0.698	
	0.720	
	0.639	
	0.732	
Behavior control	0.643	0.874
	0.783	
	0.726	
	0.804	
	0.566	
HIV prevention intentions	0.751	0.960
	0.823	
	0.858	
	0.852	
	0.756	
	0.875	
	0.794	
	0.848	
	0.830	
	0.828	

The KMO measure of sampling adequacy (MSA) results is 0.894, which indicates good partial correlation exhibited in the data for this study. The Bartlett's test of Sphericity result is 0.000 which means very significant. The results are shown in Table 3b.

**Table 3b:** KMO and Bartlett's test for variables.

Kaiser-Meyer-Olkin measure of sampling adequacy		0.894
	approximate chi-square	23272.476
Bartlett's test for sphericity	df	1540
	Sig.	0.000

Through confirmatory factor analysis, in the measurement model of this study, the factor loadings of the 56 items are above 0.5, which meets the standard. The combined reliability is 0.933, 0.911, 0.882, 0.830, 0.927, 0.909, 0.874, 0.878 and 0.960, respectively. The mean-variance Extraction amount (AVE) are 0.637, 0.631, 0.601, 0.496, 0.681, 0.666, 0.539, 0.595, and 0.708. All of which were greater than 0.36, indicating the validity of the pilot study questionnaire was good. Table 3c indicates the results of Variable Convergent Validity.

**Table 3c:** Variable convergent validity test.

	**Combined**	**Mean variance**
**Variable**	**reliability (CR)**	**extraction (AVE)**
Mindfulness	0.933	0.637
Culture	0.911	0.631
Perceived benefits	0.882	0.601
Perceived barriers	0.830	0.496
Self-efficacy	0.927	0.681
Attitude	0.909	0.666
Subjective Norms	0.874	0.539
Behavior control	0.878	0.595
HIV prevention intentions	0.960	0.708

The reliability and validity analysis and confirmatory molecular analysis found that demographic factors such as age and gender have a measurable impact on safe sexual behaviors and knowledge of AIDS prevention among questionnaire participants. The proportion of young people using dating apps was relatively large (69%), and the scores of young people's prevention intention and understanding of AIDS knowledge were also relatively high (M = 3.86, SD = 1.03). According to the value of the validation factor, demographic factors affect participants' HIV prevention intention; therefore, it is reasonable to set demographic factors as control variables.

The measurement of HIV-preventive intentions was adapted from the studies by Abraham et al. (1992) and Zak-Place and Stern (2004). The measurement includes the ten following items: (1) In the future, if I have sex with someone new, I will ask them about their past sexual partners. (2) In the future, if I have sex with someone new, I will ask them about their HIV testing status. (3) In the future, I intend to carry condoms if I think I might be going to have sex with someone new. (4) In the future, I intend to use a condom if I have sex with someone new. (5) In the future, I intend to get tested for HIV before having sex with someone new. (6) In the future, I intend to get tested for HIV after having sex with someone new. (7) In the future, before I have sex with someone new, I intend to require them to be tested for HIV. (8) In the future, I intend to require someone new to be tested for HIV after having sex with me. (9) If I test positive for HIV, I will not have sex with anyone. (10) If someone tests positive for HIV, I will not have sex with them. The Cronbach's alpha is 0.927.

#### Mindfulness and HIV prevention intentions

Mindfulness was measured using the 15-item mindful attention and intention scale (MacKillop and Anderson, 2007), with higher scores indicating greater mindfulness. In this study, we used eight items among them to investigate participants' mindfulness. The items (Cronbach's alpha = 0.933) are as follows: (1) I can experience an emotion and not be conscious of it until later. (2) I break or spill things because I am being careless, not paying attention, or thinking about something else. (3) I find it difficult to stay focused on what is happening in the present. (4) I tend to walk quickly to where I'm going without paying attention. (5) I tend not to notice feelings of physical tension or discomfort until they really grab my attention. (6) When I learn a new person's name, I forget it as soon as I have been told it. (7) I feel like I am “running on automatic” without paying much attention to what I am doing. (8) I rush through activities without being attentive to them. The results of the hypothesis testing are shown in Table 4. It was found that mindfulness had a positive and significant effect on users' HIV-preventive intentions (β = 0.216, *p* = 0.001). Thus, H_1_ is supported.

**Table 4:** Hypothesis testing.

**Variable path**	**Standardized parameter estimate β**	**S.E**.	**C.R**.	**P**	**Hypothesis test results**
Mindfulness = >HIV prevention intentions	0.216	0.056	3.293	0.001	Support H1
Culture = >HIV prevention intentions	0.112	0.029	2.983	0.004	Support H2
Perceived benefits = >HIV prevention intentions	0.285	0.040	5.975	***	Support H3
Perceived barriers = >HIV prevention intentions	−0.117	0.029	−2.957	0.003	Support H4
Self-efficacy = >HIV prevention intentions	0.198	0.063	3.115	0.002	Support H5

^***^ Means *p* < 0.001.

This result corresponds with a study of users' HIV preventive perceptions that used the HBM (Liu H. et al., 2022). Moreover, the findings are consistent with those of previous studies by Vallejo and Amaro (2009) and Kerrigan et al. (2021), which found that mindfulness interventions are an important factor in improving mental health and HIV outcomes among female sex workers. In addition, Yang et al. (2015) suggested that prevention messages using mindlessness concepts should be developed in collaboration with the transmission of blood-borne viruses among people who inject drugs. Cluver et al. (2022) also mentioned that mindfulness significantly affects the integration of mental health care into HIV services, community services, and family services for adolescents living with HIV. Furthermore, previous research on mindfulness strongly predicted the essential factors of HIV prevention through models of nursing care (Relf, 2022). The “mindful rational living” approach has been proven to incorporate mindfulness techniques with rational emotive behavioral therapy strategies to address HIV in school settings (Chenneville and John Walsh, 2016). This finding is consistent with that of the study conducted by Koo et al. (2014), which clarified the relationship between mindfulness and HIV risk behaviors in college students. Therefore, a high degree of mindfulness influenced dating app users' awareness of HIV prevention.

#### Culture and HIV prevention intentions

Culture has been defined as a unique meaning and information system that is shared by a group and transmitted across generations, allowing the group to meet its basic survival needs, pursue happiness and wellbeing, and derive meaning from life (Matsumoto, 2007). It has further been categorized into two main dimensions: collectivism and individualism (Singelis et al., 1995). Based on these studies, we designed six questions (Cronbach's alpha = 0.909) to measure people's mindfulness and traditional cultural factors. These questions are as follows: Do my parents think I am bad when I use Hello Group? Do my friends think I am bad when I use Hello Group? Does my spouse think I'm bad when I use Hello Group? Will my use of Hello Group affect my relationship with my parents? Will my use of Hello Group affect my relationship with my friends? Will my use of Hello Group affect my relationship with my spouse?

According to the hypothesis testing results shown in Table 4, Chinese culture had a positive and significant effect on users' HIV-preventive intentions (β = 0.112, *p* = 0.004). This can be interpreted to mean that most users are deeply influenced by traditional Chinese culture, especially the value of collectivism. Users of online dating apps still attach importance to their relationships with family and friends; therefore, it is necessary to raise HIV-preventive intentions during online dating. Therefore, H_2_ was supported.

This finding is in line with previous research that found that culture plays an important role in solving the problem of HIV prevention among young people (Lillie, 2006). Weeks et al. (2010), Le et al. (2018), and Bond and Ramos (2019) also mentioned that the subject matter was culturally relevant and focused on the topic of female-controlled HIV prevention methods. Full use of peer education and social interaction-based interventions may help prevent and control the spread of HIV and AIDS among female sex workers in China (Dong et al., 2019). In addition, some scholars believe that HIV and STI prevention programs can address the cultural, social, and economic constraints facing the migrant population in China (Hong et al., 2006). A study by Zhang et al. (2019), which examined the preferences of MSM for an HIV prevention mobile phone app, also supported this finding. Then, under cultural and family pressure (the Chinese traditional moral code, family values, and gender roles), Jones (1999) also examined the concept of “culture” and its relationship to HIV prevention. Johns found that culture influenced dating app users' awareness of HIV prevention.

#### Perceived benefits and HIV prevention intentions

In our model, “perceived benefits” refer to the perceptions of positive outcomes associated with users' HIV-preventive intentions. This study used five items that were outlined by Finkel et al. (2012) to measure users' perceived benefits. The following items (Cronbach's alpha = 0.909) were screened out: (1) Compared with traditional dating services, I am able to access a larger number of potential partners on Hello Group, (2) Compared with traditional dating services, Hello Group allows me to be accessible to more potential partners, (3) Compared with traditional dating services, Hello Group allows me to overcome time and place limitations when interacting with potential partners, (4) Compared with traditional dating services, on Hello Group, I am more able to enter my own search criteria when seeking out partners, (5) Compared with traditional dating services, Hello Group allows potential partners to be more easily matched to me. According to the hypothesis testing results shown in Table 4, perceived benefits had a positive and significant effect on users' HIV-preventive intentions (β = 0.285, *p* < 0.001). Thus, H_3_ was supported.

This outcome is consistent with a study by Fernandez et al. (2019) that found that the perceived benefits of using condoms are an important factor in raising awareness about HIV testing and prevention services. Smith et al. (2012) argue that frequent HIV testing was a perceived benefit. They also found that it is associated with HIV stigma and can enhance the reputation of pre-exposure prophylaxis users. The importance of perceived benefit was also highlighted by Fullerton (2008) study, which found that the perceived benefits of condom use had a positive effect on the acceptance of HIV. These findings are also in line with a previous study on the formation of prevention awareness by Logie et al. (2019).

Moreover, Mootz et al. (2020) proved the acceptability of using electronic healthcare predictive analytics to promote HIV prevention. Thus, perceived benefits influence the awareness of HIV prevention. The results of our study were also consistent with previous research by Isler et al. (2014), which identified minority benefits of engagement in HIV prevention research that can promote community awareness of HIV prevention. Moreover, Mutonyi and Kendrick (2010) believed that the benefits of health literacy programs in Uganda could positively influence awareness of HIV prevention among students.

#### Perceived barriers and HIV prevention intentions

We also proposed that the “perceived barriers” refer to the perceptions of negative outcomes associated with users' subjective norms and HIV prevention intentions. It has been suggested that users might hold negative attitudes toward technology with a sense of perceived risk, especially in the context of controversial technologies (Chen et al., 2021). The measurement was adapted from the studies by Finkel et al. (2012) and Chen et al. (2021). The adapted measurement included the following five items (Cronbach's alpha = 0.879): (1) When using Hello Group, I may be concerned that the platform will disclose too much information about me; (2) when using Hello Group, I may be concerned that the personal information in my profile will be misused by others; (3) the use of Hello Group may negatively affect the way others think of me; (4) the use of Hello Group may lead me be cheated; and (5) the use of Hello Group may expose me to the risk of HIV infection. The results on perceived barriers and users' HIV-prevention intentions indicated a negative relationship (β = −0.117, *p* = 0.003). This means that using dating apps may expose users to certain risks, such as personal information disclosure or HIV infection. Therefore, H_4_ was supported.

The finding is consistent with a previous study by He et al. (2017) on sociocultural barriers and negative methods of coping with AIDS in the gay community. Sutherland (2002) claimed that participation intentions and perceived barriers to online nutrition programs were influenced by knowledge, self-efficacy, and subjective norms. In this regard, perceived barriers are unique in the perceived HIV risk among African American women (Heath, 2014). In addition, this study is consistent with the previous studies which showed that perceived barriers play a role in assessing individuals' willingness to get vaccinated against COVID-19 by using the HBM and the TPB as theoretical frameworks (An et al., 2021). Furthermore, the importance of perceived barriers to HPV vaccination influences the intention to vaccinate (Nyaga, 2020). Powell and Karraker (2017) explain that parenting behavior intention is affected by barriers to parenting knowledge and subjective norms and attitudes.

#### Self-efficacy

The questionnaire investigated participants' HIV-preventive intentions and safe sexual behaviors *via* the following six survey items (Cronbach's alpha = 0.927): (1) I am confident that it is best to use condoms during sexual behavior; (2) I negotiate with netizens before engaging in sexual behavior; (3) I avoid engaging in sexual acts with drug addicts; (4) I test myself after engaging in dangerous sexual behavior; (5) I keep using condoms during sexual acts; and (6) I frequently use condoms during sexual acts with strangers. According to the hypothesis testing results, self-efficacy has a positive and significant effect on users' HIV-preventive intentions (β = 0.198, *p* = 0.002). Therefore, H_5_ was supported.

This study is also consistent with a study that found that self-efficacy is associated with introducing free antiretroviral therapy (Makishe, 2013). In addition, middle-class African American women's attitudes, beliefs, perceptions, and behaviors related to HIV risk are influenced by social and cultural norms and self-efficacy (Heath, 2014). In China, the self-efficacy of minority groups strongly influences the awareness of HIV prevention (Dai, 2018). The self-efficacy of laborers was effective at increasing HIV knowledge and decreasing HIV risk behaviors (Dong et al., 2019). Buseh et al. (2006) also mentioned that condom use self-efficacy affected HIV-protective intentions.

So far, we have discussed the direct effects of mindfulness, culture, perceived benefits, perceived barriers, and self-efficacy on users' HIV prevention intentions, and all the hypotheses were supported. In the following section, we will discuss the mediating effects of each variable.

### Indirect effects

#### Measurement of attitude, subjective norms, and behavior control

Several studies demonstrated the relationship between attitudes and HIV-preventive intentions (Zak-Place and Stern, 2004; Traube et al., 2011; Qiu and Huang, 2020). In this study, the following five items (Cronbach's alpha = 0.907) were used to measure users' attitudes toward HIV: (1) Nobody deserves to be HIV-positive; (2) people with HIV have nothing to feel guilty about; (3) it is safe for people with HIV to work with children; (4) people with HIV are no different from anybody else; and (5) the needs of people with HIV should be prioritized (Green, 1995). In research by Ross and Mclaws (1992) and Smerecnik and Ruiter (2010), the following items were used to measure users' subjective norms: (1) Most people who are important to me think that I should use a condom; (2) all my friends think that I should use a condom; (3) casual sexual partners think I should use a condom during unsafe sex; (4) I generally observe my casual sexual partners closely; (5) partners in the Hello Group think that I should use a condom; and (6) I generally closely observe my partners in the Hello Group (Cronbach's alpha = 0.868). The items for measuring behavior come from Michael and Monk's study (Michael Monk, 2012), including the following statements: (1) I reduced the frequency of intercourse; (2) I decreased my number of sex partners; (3) I reduced my frequency of using the Hello Group app; (4) I used condoms when I had sex; and (5) I got tested for HIV (Cronbach's alpha = 0.874).

#### Mediation effect analysis

This study added attitude, subjective norms, and behavior control as mediating variables between the five independent variables (mindfulness, culture, perceived benefits, perceived barriers, and self-efficacy) and the dependent variables (HIV prevention intentions). It can be seen from Table 5 that users' subjective norms and behavior control toward sexual acts have obvious mediating effects between their mindfulness, attitude, culture, perceived benefits, perceived barriers, self-efficacy, and HIV prevention intention. The number of repeated samplings for all samples is 520, and the confidence interval is set at 95%. Table 5 shows the results.

**Table 5:** Standardized Bootstrap mediation test results.

**Path**	**Direct effect value**	**Mediation effect value**	**Total effect value**	**Hypothesis test results**
Mindfulness,	0.173^**^	0.189^**^	0.216^**^	Support H6
Culture,	0.097^*^	0.101^**^	0.112^**^	Support H7
Perceived Benefits = > Attitude = > HIV Prevention Intentions	0.082	0.053	0.285^**^	Not support H8
Perceived Benefits,	0.262^**^	0.172^**^	0.285^**^	Support H9
Perceived Barriers = > Subjective norms = > HIV Prevention Intentions	−0.101^**^	0.121^**^	−0.117^**^	Support H10
Self-efficacy = > Behavior control = > HIV Prevention Intentions	0.149^**^	0.133^**^	0.198^**^	Support H11

^*^ Means *p* < 0.05, ^**^ means *p* < 0.01.

When mindfulness, perceived benefits, self-efficacy, attitude, subjective norms, and behavior control were used to predict HIV prevention intention simultaneously, attitude (β = 0.263, *p* < 0.01), subjective norms (β = 0.384, *p* < 0.01), and behavior control (β = 0.241, *p* < 0.01) had a significant and positive impact on HIV prevention.

However, when using perceived barriers, attitude and subjective norms, and behavior control to predict HIV prevention intentions, perceived barriers had a negative impact on HIV-preventive intentions (β = −0.101, *p* < 0.01). This suggests that it should enable the participants to shed their existing misconceptions in each node of the network agreement and improve their intentions for HIV prevention.

To explore the mediating effect between the users' attitude toward HIV, subjective norms, and behavior control of the five independent variables—mindfulness, culture, perceived benefits, perceived barriers, self-efficacy, and the users' intention of HIV prevention—this study used the bootstrap method. Attitude mediates the relationship between mindfulness and intentions (β = 0.189, *p* < 0.01) and the relationship between culture and intentions (β = 0.101, *p* < 0.01). Therefore, H_6_ and H_7_ were supported.

Zang et al. (2014) believed that HIV stigma might mediate the relationship between collectivist culture and social network support, providing an empirical basis for interventions that include aspects of culture in HIV intervention strategies. Sexuality, condoms, and drugs are sensitive topics in Vietnamese culture, especially when men and women communicate with each other (Van Nguyen et al., 2013). Sanchez et al. (2016) also established the efficacy of SEPA, a CDC evidence-based and culturally tailored HIV risk reduction intervention practiced among Latina immigrants in the farmworker community. In addition, He et al. (2017) found that sociocultural factors influence gay men's sexual beliefs and behaviors in contemporary China; they also analyzed the implications of this finding for the HIV epidemic. Having a cohesive family (a collective culture that values family relationships), a strong social network, and peers who encourage healthy behaviors and discussions of sexual health help Vietnamese adolescents achieve a higher level of HIV knowledge (Nguyen et al., 2015). Therefore, H_7_ was supported.

However, the mediating effect of attitude on perceived benefits and intentions is insignificant (*p* > 0.05). Therefore, H_8_ was not supported. This conclusion differs from previous studies. Morar et al. (2018) mentioned that positive attitudes toward HIV prevention mediate the relationship between condom use and the benefits of active testing on peer prevention behavior. Attitude has a mediating effect on the perceived benefits of HIV prevention services and prevention awareness (Otengah and Omolo, 2020). Kim (1996) maintained that the benefits of condom use had increased HIV awareness and prevention knowledge among Korean Americans. In line with the TPB, participants' attitudes and self-efficacy toward the course were significant and meaningful predictors of their learning intention and subsequent behavior change (Cooley et al., 2020). The benefits of the theater performance were described as conducive to learning, and it indicated changed attitudes and awareness toward LGBT persons and issues following a participatory theater intervention in Swaziland and Lesotho (Logie et al., 2019). Additionally, Clark et al. (2006) suggested that the perceived benefits of HIV education caused an increase in student attitudes and increased awareness of HIV prevention among young people.

Subjective norms mediate the relationship between perceived benefits and intentions (β = 0.172, *p* < 0.01) and the relationship between perceived barriers and intentions (β = 0.121, *p* < 0.01). Therefore, H_9_ and H_10_ were supported. Previous studies by Du et al. (2020) showed that facilitating factors, such as HIV prevention awareness, may be supported by subjective norms and the perceived benefits of taking HIV antiretroviral therapy. Several studies related to HIV prevention awareness showed that perceived benefits are effectively influenced by subjective norms (Glick and Sahn, 2007; Cui et al., 2022). Alzahrani and Daim (2019) explain that, among patients with AIDS, the adoption and use of and perceived benefits of tethered electronic personal health records were indirectly affected by subjective norms. In addition, this study is also consistent with research on pregnant Ghanaian women's knowledge, attitudes, and intentions regarding voluntary prenatal testing for HIV (Lee, 2003). UMUHIRE (2020) proposed that subjective norms, perceived benefits, and attitudes positively influenced women's use of contraceptive methods to prevent unwanted pregnancies. In this regard, non-adherence across multiple therapeutic classes among the elderly is based on the influence of perceived benefits and subjective norms (Ding, 2010). Thus, the current study supported this hypothesis.

Moreover, H_9_ was supported. An et al. (2021) stated that, for the HBM and TPB constructs, respondents were more likely to accept vaccination if they had a higher level of cues to action and self-efficacy and a lower level of perceived barriers. In addition, this study is also inconsistent with a previous study by Tweed (2008) that emphasizes the role of perceived barriers and highlights that the relationships between perceived barriers to exercise and determinants of physical activity are indirectly influenced by subject norms. Smith (2018) examined the interaction of clinicians' subjective norms and perceived clinical screenings for depression disorders among older adults to influence clinical behavior. Consumer rejection of the practice of coupon use is influenced by attitudes, subjective norms, and perceived barriers (Andrews, 2016). These studies also align with our conclusions. Therefore, H_10_ was supported.

In addition, behavior control has a significant mediating effect on self-efficacy and intentions (β = 0.133, *p* < 0.01). Previous research demonstrated that behavioral control motivates HIV prevention awareness. When people use HIV prevention services, they tend to maintain strong self-efficacy, which is mediated by education and behavioral control (Omolo, 2018). Zhao et al. (2020) believe that people with higher self-efficacy in China were associated with HIV-related behaviors (history of STI testing) and influenced by personal behavior control. Many studies in the field of health communication found that the ability to correctly use available services is enhanced by knowledge and behavior control, which have an impact on HIV prevention and treatment interventions (Fishbein and Cappella, 2006; Makishe, 2013; Chimoyi et al., 2022). Shongwe (2014) also claimed that young peoples' intentions to adopt a mobile health information system implemented as an HIV information dissemination tool were influenced by behavioral control. Actual control, self-efficacy of condom use, and the intention-to-behavior relationship with AIDS were important moderators (Dai and Harrington, 2021). Thus, H_11_ was also supported.

## Conclusions

Due to the influence of traditional culture and public values, hookups have a low level of social acceptance in China, and it is more common to ask strangers through online social apps. This has also led to a lack of attention and research on hookup culture in China. Online dating applications, such as Hello Group, allow mobile users to meet potential partners through social media, causing more radical attitudes among Chinese people toward sex. Although these apps may satisfy users' needs for love and sex, the associated risks are often overlooked. Indeed, the risk of HIV infection should be considered when deciding whether to arrange a meet-up with a stranger.

This study investigated the influencing factors related to hookups in the context of the Hello Group app and HIV prevention intentions. The results showed that mindfulness, the Chinese cultural context, perceived benefits, and self-efficacy were the main predictors of users' HIV-protective intentions when using online dating apps. Among the HIV-related perceived risks of mobile dating, only perceived barriers have a negative effect on users' HIV-preventive intentions. In addition, attitude, subjective norms, and behavior control act as mediating variables between independent variables and HIV-protective intention; however, the mediating effect of attitude on perceived benefits and intentions is insignificant.

The main contributions of this study are as follows: First, most existing studies that employed qualitative methods were case studies. This study used quantitative research methods and complemented studies on groups that engage in hookups. For this study, we collected a large amount of data through online questionnaires, used SPSS software and SEM to analyze the data, drew a portrait of groups of Chinese people who engage in hookups, and corrected their perception of HIV risk while increasing their HIV protection intention. Second, this study combined the HBM with the TPB and incorporated the connection between traditional culture and mindfulness for users' HIV prevention intentions. The study found that users are afraid of unsafe sex and believe that safe sex can effectively reduce their risk of HIV infection; therefore, they are more likely to adopt safe sex practices. Mindfulness not only relieves the psychological discomfort of patients with AIDS but also improves their HIV prevention intentions. The study also found that some users have misconceptions regarding HIV risks. Without condoms, one can gain the trust of one's partner, use testing to prevent AIDS, and more. These actions increase the risk of AIDS infection among people who engage in hookups. As a first step, those interested in online dating should correct the common misunderstandings that exist at every stage of the process, focus on public awareness campaigns and sexual health education messaging, spread the word about drug prevention before and after AIDS exposure, fortify psychological counseling, enhance relevant legal systems, standardize the development of online dating applications, contain the outbreak at its source, sever all possible avenues of transmission, and thereby effectively halt the disease's spread.

This study also has certain limitations because there are many dating apps in China and because users' characteristics vary according to each app. This study only investigated the users of the app Hello Group. Due to privacy concerns regarding questionnaire participants and the lack of trust in cyberspace, it is difficult to collect survey data; therefore, our sample size was small. In addition, the hypothesis model mainly emphasizes the rational factors of engagement behavior; however, in some situations, HIV prevention behavior is affected and controlled by other factors, such as personal habits, moral norms, social identity cognition, emotional factors, and other irrational factors, all of which can determine people's behavior choices in different situations. Therefore, in future research, other factors should be considered to study the HIV-preventive intentions of hookup app users. In addition, other methods to enrich the collection of relevant data should be considered to ensure the accuracy and representativeness of the data. This will ensure that the research is more meaningful and can contribute to preventing HIV transmission.

## Data availability statement

The original contributions presented in the study are included in the article/supplementary material, further inquiries can be directed to the corresponding author/s.

## Ethics statement

Ethical review and approval was not required for the study on human participants in accordance with the local legislation and institutional requirements. Written informed consent from the patients/participants or patients/participants legal guardian/next of kin was not required to participate in this study in accordance with the national legislation and the institutional requirements.

## Author contributions

ML and NL: conceptualization, methodology, and survey and data analysis. ML: coding and writing—original draft preparation. NL: supervision and writing—review and editing. All authors have read and agreed to the published version of the manuscript.
