# Corrigendum: A study of the factors influencing HIV-preventive intentions among “hookup” application users

**DOI:** 10.3389/fpsyg.2023.1166258

**Published:** 2023-02-28

**Authors:** Mengyu Li, Ning Li

**Affiliations:** ^1^College of Humanities and Development Studies, China Agricultural University, Beijing, China; ^2^Faculty of Modern Languages and Communication, Universiti Putra Malaysia, Serdang, Selangor, Malaysia; ^3^School of Journalism and Communication, Zhengzhou University, Zhengzhou, China; ^4^Department of Media and Communication, Kangwon National University, Chuncheon-si, South Korea

**Keywords:** social app, health belief model, planned behavior theory, hooking up, mindfulness, SEM model, HIV prevention

In the published article, there was an error in the author list. Mengyu Li and Ning Li should share first authorship.

The authors apologize for this error and state that this does not change the scientific conclusions of the article in any way. The original article has been updated.

